# Endothelial progenitor cell transplantation attenuates lipopolysaccharide-induced acute lung injury via regulating miR-10a/b-5p

**DOI:** 10.1186/s12944-019-1079-3

**Published:** 2019-06-07

**Authors:** Yan Jin, Chen Yang, Xintong Sui, Quan Cai, Liang Guo, Zhi Liu

**Affiliations:** Department of Emergency, The First Affiliated Hospital of China Medical University, No.155 Nanjing North Street, Heping District, Shenyang, Liaoning Province 110001 People’s Republic of China

**Keywords:** Acute lung injury, Endothelial progenitor cells, microRNA, Cell transplantation, Neovascularization

## Abstract

**Background:**

Bone marrow-derived endothelial progenitor cells (EPCs) are shown to attenuate lipopolysaccharide- (LPS-) induced acute lung injury (ALI) in animal models. However, the molecular mechanism is largely unknown.

**Materials and methods:**

The animal model of ALI was induced by intratracheal instillation of purified LPS with 2.5 mg/ml/kg. The expression of microRNAs and ADAM15 in lung tissues and LPS-induced mouse pulmonary microvascular endothelial cells (MPMVECs) was determined by quantitative real-time PCR and western blot analysis. The target relationship between miR-10a/b-5p and ADAM15 was confirmed by luciferase reporter assay and RNA interference. The effect of EPCs on MPMVEC proliferation was detected by MTT assay.

**Results:**

EPCs increased the expression of miR-10a/b-5p and reduced ADAM15 protein level in LPS-induced ALI lung tissues and MPMVECs (*p* < 0.05), and promoted LPS-induced MPMVEC proliferation (*p* < 0.05). ADAM15 was confirmed to be a downstream target of miR-10a/b-5p. Additionally, EPCs promoted LPS-induced MPMVEC proliferation and exerted the therapeutic effect of ALI via regulating miR-10a/b-5p/ADAM15 axis.

**Conclusion:**

EPC transplantation exerted its therapeutic effect of ALI via increasing miR-10a/b-5p and reducing ADAM15, thus providing a novel insight into the molecular mechanism of EPC transplantation in treating ALI.

**Electronic supplementary material:**

The online version of this article (10.1186/s12944-019-1079-3) contains supplementary material, which is available to authorized users.

## Introduction

Sepsis, characterized by the activation of inflammation and coagulation and inhibition of fibrinolysis, is a major public health problem worldwide. With a high mortality and an increasing incidence, sepsis is becoming the leading cause of intensive care unit (ICU) mortality in China, with the mortality rate of 28.7% for severe sepsis and 33.5% for septic shock [1]. Acute lung injury (ALI) is primarily caused by sepsis concerning both pulmonary and nonpulmonary infections, and is characterized by tachypnea and hypoxemia, which often progresses to respiratory failure requiring intubation and mechanical ventilation [2]. The pathological mechanisms of ALI involve in the endothelial dysfunction as well as the increased vascular permeability [3]. Monocytes, tissue macrophages, other myeloid-derived cells, and to some extent endothelial cells, are the cornerstones of the innate immune response. Lipopolysaccharide (LPS), known as endotoxin, and/or other pathogen-associated properties activate pathogen recognition receptors on endothelial cells and other cells, leading to the release of inflammatory mediators and tissue factors, and resulting in the initiation of inflammatory and coagulation cascades [4, 5]. Therefore, restoring endothelial function and vascular permeability is the key point of treating sepsis and ALI. However, the specific molecular mechanism of regulating endothelial function remains unknown.

Bone marrow-derived endothelial progenitor cells (EPCs), shown to participate in physiologic and pathologic neovascularization, have been investigated as therapeutic agents in vascular-associated diseases [6, 7]. EPC transplantation thus constitutes a novel therapeutic strategy that could provide a robust source of viable endothelial cells to supplement the contribution of endothelial cells resident in the adult vasculature [8]. It has been reported that EPC transplantation can significantly reduce lung injury in septic mice and rats [9–11]. In addition, EPC transplantation has been demonstrated to attenuate LPS-induced lung injury in the animal models [12, 13], although little is known about the underlying mechanisms.

ADAM15, belonging to the ADAMs (a disintegrin and metalloproteinase), is found in human vein endothelium and is associated with certain types of cancer and chronic inflammatory or immunological disorders [14]. Previous studies have demonstrated that ADAM15 regulates endothelial permeability, and contributes to atherosclerosis by mediating endothelial barrier dysfunction [14, 15]. In LPS-induced inflammatory injury, knockdown of ADAM15 attenuates pulmonary hyperpermeability and ALI, indicating ADAM15 as a pro-inflammatory protein in ALI [16].

MicroRNAs (miRNAs), a class of non-coding RNAs, have been reported to play an important role in cellular development, genomic imprinting, and regulating cellular functions [17]. For instance, miR-15a, miR-34a, miR-155, and miR-146a have been demonstrated to have aberrant plasma levels in sepsis [18]. Moreover, miR-10 is reported to regulate human endothelial cells during angiogenesis [19]. Hence, in this current study, we explored the expression of different miRNAs in the ALI mouse model, and further investigated the molecular mechanism of EPC transplantation in treating LPS-induced ALI.

## Materials and methods

### Animals

A total of 48 male C57BL/6 mice ranging from 4 to 6 weeks old were purchased from the Animal Experimental Center, Guangdong Academy of Medical Science (Guangdong, China). Animals were raised and used in accordance with the National Institutes of Health Guidelines on the Care and Use of Laboratory Animals. All experimental procedures performed were approved by the First Affiliated Hospital of China Medical University Committee on Animal Care.

### Animal model

The animal model of ALI was induced by intratracheal (IT) instillation of purified lipopolysaccharide (LPS) with 2.5 mg/ml/kg (*Escherichia coli* 055:B5, Solarbio, Beijing, China) as described [13]. Briefly, mice were anesthetized with intraperitoneal (IP) injection of sodium pentobarbital (60 mg/kg). EPCs (3 × 10^6^ cells in 100 ml phosphate buffer saline [PBS]) or PBS were intravenously (IV) injected 2 h after LPS-induced ALI. After the IV injection of EPCs or PBS, they were assigned to 3 groups: sham group (*n* = 6), ALI + PBS group (*n* = 6), and ALI + EPCs group (*n* = 6). Mice were sacrificed at 48 h after IT LPS administration and samples of lung tissue were collected. To down-regulate the expression of miR-10a/b-5p in the animal model, the IT administration of miR-10a/b-5p inhibitor or control inhibitor (2 mg/kg) was performed 24 h before the IT instillation of LPS.

### Isolation and culturing of EPCs

Bone marrow-derived mononuclear cells were obtained from femurs and tibias of mice and isolated by density-gradient centrifugation with Histopaque 1.083 (Sigma-Aldrich, Shanghai, China). Mononuclear cells were washed and plated on culture dishes coated with human plasma fibronectin (T&L Biological Technology, Beijing, China) and cultured in endothelial cell growth medium-2-microvascular (EGM-2-MV) (Lonza, Nanjing, China) supplemented with EGM-2-MV SingleQuots and mouse recombinant vascular endothelial growth factor, epidermal growth factor, fibroblast growth factor, and insulin-like growth factor (ThermoFisher, Shanghai, China). The adherent cells at day 14 were harvested by trypsinization for the animal treatment studies.

### EPC conditioned medium preparation

EPC conditioned medium (EPC-CM) was prepared as previously described [20]. Briefly, EPCs were plated at 10% confluence and cultured for 5 days on type 1 collagen in room air in complete EGM-2-MV medium (Lonza). Cells were rinsed twice with PBS at day 5, and the medium was replaced with Dulbecco’s modified Eagle’s medium (DMEM, Invitrogen, Shanghai, China) with 2.5% FBS. EPC-CM was collected after 24 h, and was filtered with 0.2 μM filters and frozen at − 70 °C until use. The unconditioned control medium was treated similarly, but never exposed to EPCs.

### Co-culturing with EPCs

Mouse pulmonary microvascular endothelial cells (MPMVECs) were obtained from the Procell Life Science & Technology (Wuhan, China). The MPMVECs were cultured in the EPC-CM for 24 h followed by the stimulation of LPS (500 ng/ml) for another 24 h.

### Cell transfection

The miR-10a/b-5p inhibitor/negative control (NC), miR-10a/b-5p mimic/pre-NC, and small interfering- (si-) ADAM15/si-control were synthesized by Gene Pharma (Shanghai, China). The MPMVECs were seeded in six-well plates and cultured in DMEM containing 10% fetal calf serum, 100 U/mL penicillin, and 100 mg/mL of streptomycin at 5% CO_2_ and 37 °C. Lipofectamine 2000 (Invitrogen, San Diego, CA, USA) was used in accordance with the manufacturer’s instructions. After the transfection, MPMVECs were co-cultured with EPCs followed by the stimulation of LPS.

### Quantitative real-time PCR

To determine the expression of miR-10a/b-5p and mRNA of ADAM15, total RNA was isolated by TRIzol reagent (Invitrogen). The reverse transcription was performed to synthesize cDNA via a reverse transcription kit (Takara, Dalian, China). Quantitative real-time PCR (qRT-PCR) was conducted using the SYBR Green PCR Kit (Takara) on the Applied Biosystems 7500 qRT-PCR System (Applied Biosystems, Foster City, Calif, USA), and U6 was used as the internal control. The expression of RNAs was calculated by 2^–ΔΔCT^ method. The primer sequences were as follows:

ADAM15: Forward 5′-GGCTGGCAGTGTCGTCCTACCAGAGGG-3′.

Reverse 5′-GGTGCACCCAGCTGCAGTTCAGCTCAAGTCC-3′.

miR-10a-5p: Forward 5′-CGCTACCCTGTAGATCCGAA-3′.

Reverse 5′-GTGCAGGGTCCGAGGT-3′.

miR-10b-5p: Forward 5′-CGGCGGATACCCTGTAGAAC-3′.

Reverse 5′-GGCTGTCGTGGACTGCG-3′.

U6: Forward 5′ -CTCGCTTCGGCAGCACA-3′.

Reverse 5′-AACGCTTCACGAATTTGCGT-3′.

### Western blot analysis

Total protein was extracted via cell lysed in RIPA lysis buffer containing a protease inhibitor cocktail and a Halt phosphatase inhibitor (Thermo Scientific, Shanghai, China) and centrifugation. Following a 10% SDS-PAGE gel fractionation and membrane transference, the blots were transferred to the PVDF and maintained with primary antibodies against ADAM15 (1:500, Abcam, London, UK), or β-actin (1:1000, Abcam) at 4 °C for 24 h. Then the PVDF was incubated with secondary antibodies at room temperature for 1 h. Specific bands were measured using the ECL system on the Bio-Rad electrophoresis image analyzer (Bio-Rad, Hercules, CA, USA).

### Lung wet-to-dry ratio and lung injury score

The right lung was excised, dried gently with a blotting paper, and weighed. The right lung was then dried at 80 °C for 72 h and reweighed. The ratio of wet-to-dry weight was used as the degree of lung edema. The other part of lung tissue was fixed by 100 g/l formaldehyde and was cut into olefin slice followed by being evaluated via the microscope. The lung injury score (LIS) was determined as described [21]: (i) the severity of leukocytes sequestration in the lung tissue: 0 = 0%, 1 = 0–25%, 2 = 25–50%, 3 = 50–5%, 4 = 75–100%; (ii) the severity of leukocytes sequestration in lung alveolus: 0 = none, 1 = few, 2 = a lot of, 3 = almost full, 4 = absolutely full; (iii) the severity of exudation (such as fibrin, transparent membrane, edema liquor) in lung alveolus: 0 = none, 1 = few, 2 = a lot of, 3 = almost full, 4 = absolutely full; and (iv) the pulmonary interstitial thickness: 0 = < 1.0 μm, 1 = 1.0 μm–2.0 μm, 2 = 2.0–3.0 μm, 3 = > 3.0 μm. The average value of 10 high power fields of view was designated as the final LIS.

### Luciferase reporter assay

The full-length ADAM15 3′-UTR was amplified by PCR and cloned into the pMIR-REPORT luciferase miRNA expression reporter vector (ThermoFisher). Site mutations were generated by PCR using the Quik-Change site-directed mutagenesis kit (Strata-gen, Santa Clara, CA, USA). For the luciferase reporter assay, 293 T cells were seeded onto 24-well plates at a density of 3 × 10^4^ cells per well and transfected with wild-type (WT) or mutated (Mut) ADAM15 3′-UTR reporter constructs (0.2 μg), miR-10a/b-5p mimic/pre-NC (50 nM), and the Renilla luciferase reporter plasmid pRL-TK (10 ng; Promega, Madison, WI, USA) using Lipofec-tamine 2000 reagent (Invitrogen). At 24 h after transfection, cells were lysed and luciferase activities were measured using the luciferase reporter assay system (Promega, Beijing, China). The firefly luciferase activity was normalized to the Renilla luciferase activity.

### 3-(4,5-dimethylthiazol-2-yl)-2,5-Diphenyltetrazolium bromide assay

Cell proliferation of MPMVECs was assessed by 3-(4,5-dimethylthiazol-2-yl)-2,5-Diphenyltetrazolium Bromide assay (MTT assay). The MPMVECs were transfected with miR-10a/b-5p inhibitor and/or si-ADAM15 followed by the co-culture with EPCs and LPS stimulation. Afterwards, the MTT assay was performed as described previously [22].

### Statistical analysis

All data were statistically analyzed by SPSS 17.0 (SPSS Inc., Chicago, IL, USA) and exhibited as means ± standard deviation (SD) with at least three repeats. Student’s t-test or an ANOVA was used to assess group difference in an appropriate pattern. *P* < 0.05 was considered as statistically significant.

## Results

### The expression of miR-10a/b-5p was increased in EPCs transplanted ALI lung tissues

**Fig. 1:**
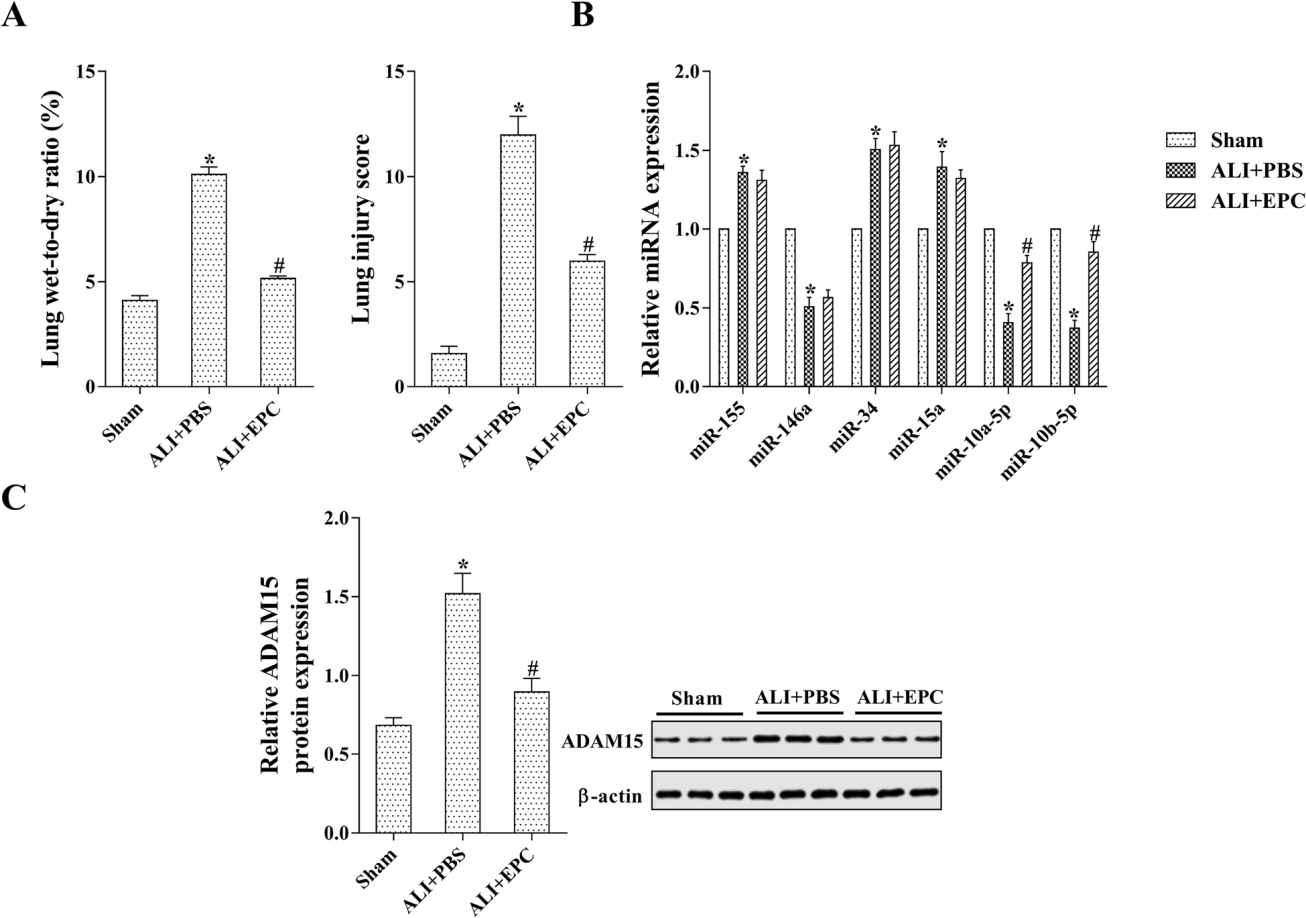
The expression of miR-10a/b-5p was increased in EPCs transplanted ALI lung tissues. The animal model of ALI was established by IT instillation of LPS in 18 male C57BL/6 mice. After the IV injection of EPCs or PBS, they were assigned to 3 groups: sham group (*n* = 6), ALI + PBS group (*n* = 6), and ALI + EPCs group (*n* = 6). **a**: The level of lung wet-to-dry ratio and the average value of LIS. **b**: The expression of ALI-related miRNAs was detected by qRT-PCR. **c**: The expression of ADAM15 was detected by qRT-PCR and western blot analysis. *p < 0.05 vs. sham; #*p* < 0.05 vs. ALI + PBS

It has been reported that EPC transplantation can significantly reduce lung injury in septic mice and rats [9–11]. We first established the animal model of ALI by IT instillation of purified LPS in mice. The lung wet-to-dry ratio and the LIS in the ALI + EPCs group were dramatically lower than that in the ALI + PBS group, indicating the relief of lung injury after EPC transplantation (Fig. 1a, *p* < 0.05). We detected the serum level of IL-6 and TNF-α, which are both inflammatory factors, by using ELISA assays, and the pulmonary tissue level of HMGB1, which is an inflammation-related protein, by using western blot analysis. As shown in Additional file 1: Figure S1, the increase in IL-6, TNF-α, and HMGB1 levels after ALI was attenuated by EPC transplantation. These data revealed the anti-inflammation function of EPC transplantation. We also detected the SOD level, an antioxidase, in lung tissues, and found that after EPC transplantation, the SOD level was markedly increased in comparison with the untreated group (Additional file 1: Figure S1D), indicating that EPC transplantation may exert the anti-oxidize effect via increasing SOD expression.

Several miRNAs, such as miR-155, miR-146a, miR-34, miR-15a, miR-10a-5p, and miR-10b-5p, were reported to have correlations with lung injuries [18, 19]. Therefore, the expression of these miRNAs in harvested lung tissues was determined by qRT-PCR. The result showed that the levels of these miRNAs were markedly changed in ALI lung tissues, whereas in EPCs transplanted ALI lung tissues, only the expression of miR-10a-5p and miR-10b-5p was significantly increased (Fig. 1a, *p* < 0.05). Moreover, both the mRNA and protein levels of ADAM15 were markedly enhanced by LPS administration, and these levels were decreased significantly after EPCs transplantation (Fig. 1c, *p* < 0.05).

### EPCs increased miR-10a/b-5p and reduced ADAM15 in LPS-induced MPMVECs

**Fig. 2:**
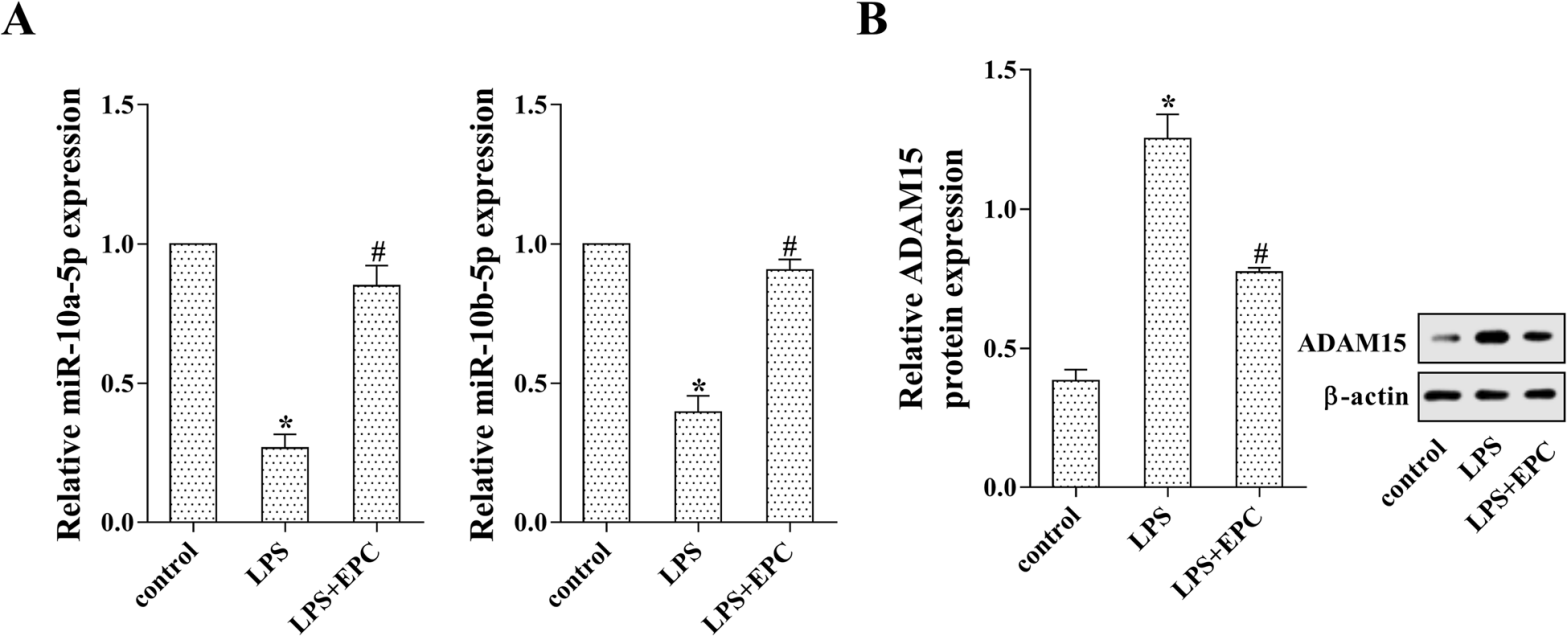
EPCs increased miR-10a/b-5p and reduced ADAM15 in LPS-induced MPMVECs. The MPMVECs were cultured in the EPC-CM for 24 h followed by the stimulation of LPS for another 24 h. **a**: The expression of miR-10a-5p and miR-10b-5p was detected by qRT-PCR. **b**: The protein level of ADAM15 was determined by western blot analysis. *p < 0.05 vs. control; #*p* < 0.05 vs. LPS

The MPMVECs were co-cultured with EPCs for 24 h followed by the stimulation of LPS. The expression of miR-10a-5p and miR-10b-5p was markedly reduced after LPS stimulation; however, the MPMVECs co-cultured with EPCs appeared a significant increase in the expression of miR-10a-5p and miR-10b-5p (Fig. 2a, *p* < 0.05). Additionally, the protein level of ADAM15 was dramatically increased by LPS stimulation, whereas it was down-regulated by EPCs (Fig. 2b, *p* < 0.05). These data demonstrated that EPCs increased the expression of miR-10a/b-5p and reduced the protein level of ADAM15 in LPS-induced MPMVECs.

### EPCs promoted LPS-induced MPMVEC proliferation via miR-10a/b-5p

**Fig. 3:**
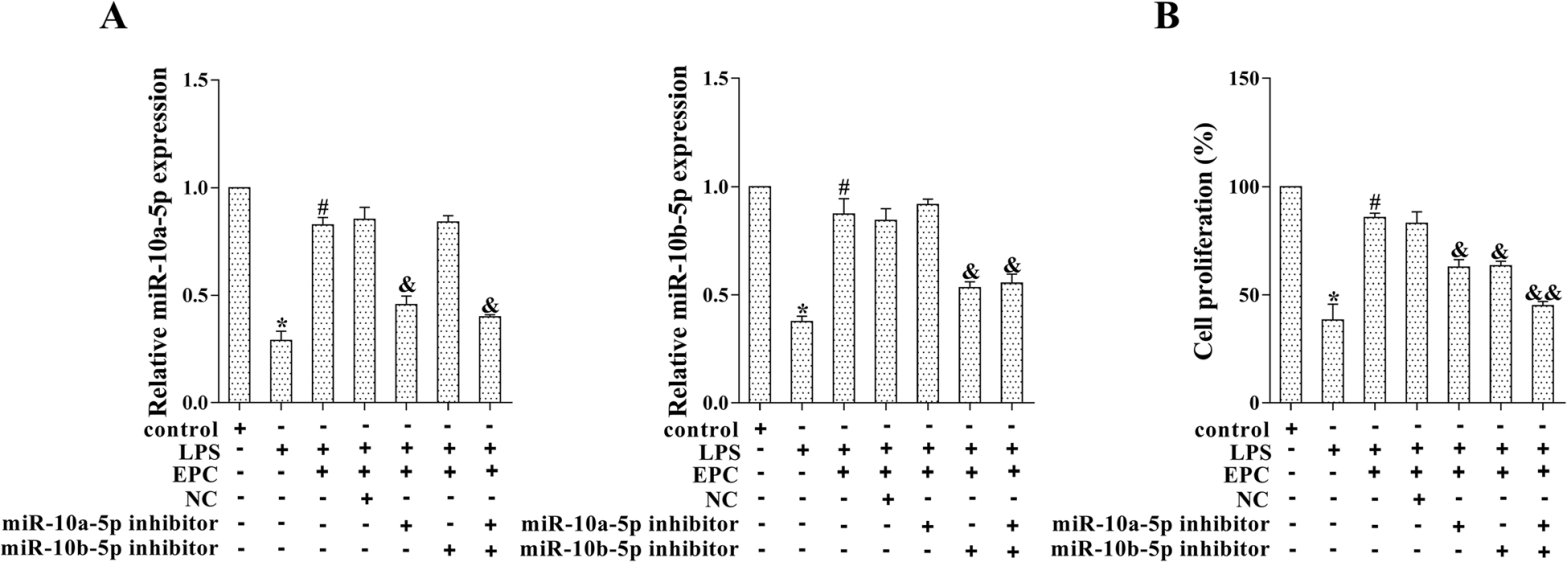
EPCs promoted LPS-induced MPMVEC proliferation via miR-10a/b-5p. The MPMVECs were transfected with miR-10a-5p inhibitor and/or miR-10b-5p inhibitor followed by the co-culture with EPCs and LPS stimulation. **a**: The expression of miR-10a/b-5p was detected by qRT-PCR. **b**: Cell proliferation was determined by MTT assay. *p < 0.05 vs. control; #*p* < 0.05 vs. LPS; & *P* < 0.05 vs. LPS + EPC + NC

Given the down-regulated miR-10a/b-5p in LPS-induced MPMVECs and the up-regulated miR-10a/b-5p by EPCs, we investigate the effect of EPCs on MPMVEC proliferation. The MPMVECs were transfected with miR-10a-5p inhibitor and/or miR-10b-5p inhibitor followed by the co-culture with EPCs and LPS stimulation. The result of qRT-PCR confirmed the down-regulation of miR-10a/b-5p (Fig. 3a, *p* < 0.05). Cell proliferation was determined by MTT assay. The outcome showed that MPMVEC proliferation was significantly reduced by LPS-stimulation, but it was restored by EPCs (Fig. 3b, *p* < 0.05). Such enhancement was dramatically decreased by the down-regulation of miR-10a-5p and/or miR-10b-5p; furthermore, the co-transfection with miR-10a-5p inhibitor and miR-10b-5p inhibitor appeared more reduction in cell proliferation than miR-10a-5p inhibitor or miR-10b-5p inhibitor alone (Fig. 3b, *p* < 0.05).

### ADAM15 is a downstream target of miR-10a/b-5p

**Fig. 4:**
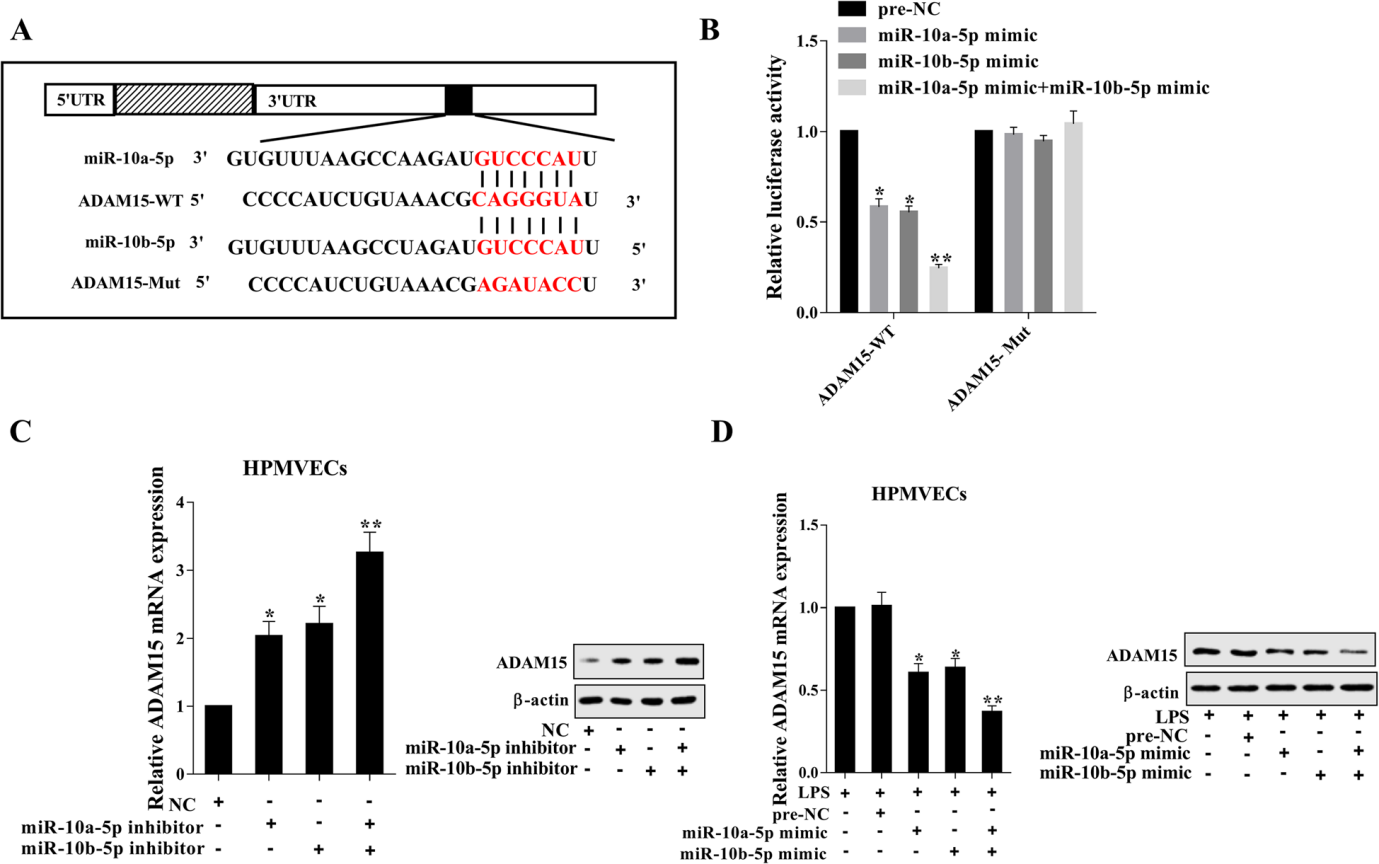
ADAM15 is a downstream target of miR-10a/b-5p. **a**: The predicted binding site of miR-10a/b-5p on ADAM15. **b**: The target relationship was confirmed by luciferase reporter assay. **c**: The mRNA and the protein level of ADAM15 in MPMVECs after miR-10a/b-5p down-regulation. **d**: The mRNA and the protein level of ADAM15 in MPMVECs after LPS stimulation and miR-10a/b-5p up-regulation. *p < 0.05 vs. NC or LPS + pre-NC; **p < 0.01 vs. LPS + pre-NC

According to the bioinformatics software (http://www.rna-society.org), miR-10a/b-5p was predicted to have the binding site on ADAM15 (Fig. 4a). We performed the luciferase reporter assay to further explore the association of miR-10a/b-5p and ADAM15. The 3′-UTR of ADAM15 was mutated as shown in Fig. 4a, and the miR-10a-5p mimic and/or miR-10b-5p mimic along with WT or Mut ADAM15 was induced into the 293 T cells. After the overexpression of miR-10a/b-5p, the luciferase activity was significantly reduced in the ADAM15-WT group in comparison with the ADAM15-Mut group (Fig. 4b, *p* < 0.05). In addition, the mRNA and the protein levels of ADAM15 were increased by miR-10a/b-5p down-regulation and were reduced by miR-10a/b-5p up-regulation and LPS stimulation (Fig. 4c and d, *p* < 0.05), and the co-transfection with miR-10a-5p and miR-10b-5p mimics/inhibitors enhanced such effect (Fig. 4c and d, *p* < 0.01). Hence, these results indicated that ADAM15 is a downstream target of miR-10a/b-5p.

### EPCs promoted LPS-induced MPMVEC proliferation via regulating miR-10a/b-5p/ADAM15

**Fig. 5:**
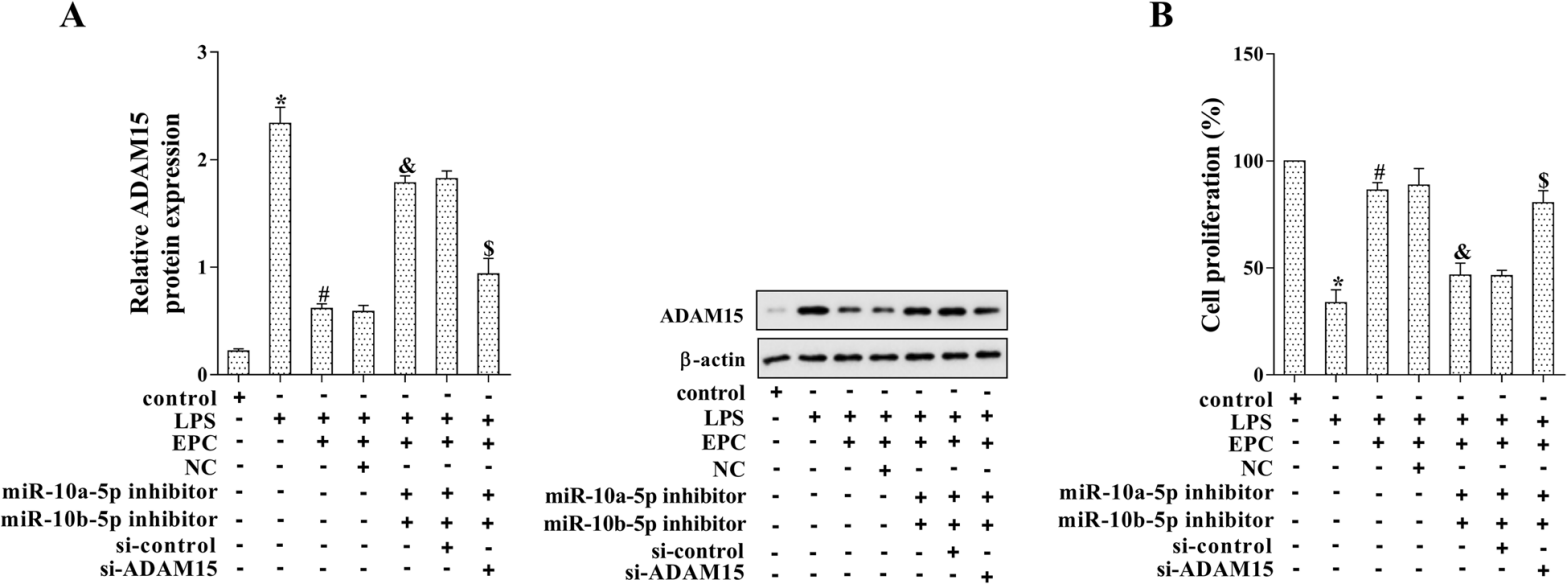
EPCs promoted LPS-induced MPMVEC proliferation via miR-10a/b-5p/ADAM15. The MPMVECs were co-transfected with miR-10a-5p inhibitor, miR-10b-5p inhibitor and si-ADAM15 followed by EPCs co-culture and LPS stimulation. **a**: The protein level of ADAM15 was determined by western blot analysis. **b**: Cell proliferation was determined by MTT assay. *p < 0.05 vs. control; #*p* < 0.05 vs. LPS; & *p* < 0.05 vs. LPS + EPC + NC; $ *p* < 0.05 vs. LPS + EPC + miR-a-5p inhibitor+miR-10b-5p inhibitor+si-control

To further investigate the modulation of EPCs on MPMVEC proliferation, MPMVECs were co-transfected with miR-10a-5p inhibitor, miR-10b-5p inhibitor and si-ADAM15 followed by EPCs co-culture and LPS stimulation. As shown in Fig. 5a, the protein level of ADAM15 was up-regulated by miR-10a/b-5p inhibitors, and was down-regulated by si-ADAM15 (*p* < 0.05). Meanwhile, si-ADAM15 restored the defect of cell proliferation caused by miR-10a/b-5p down-regulation, indicating that EPCs promoted LPS-induced MPMVEC proliferation via miR-10a/b-5p/ADAM15 pathway (Fig. 5b, *p* < 0.05).

### EPCs exerted the therapeutic effect of ALI via regulating miR-10a/b-5p/ADAM15

**Fig. 6:**
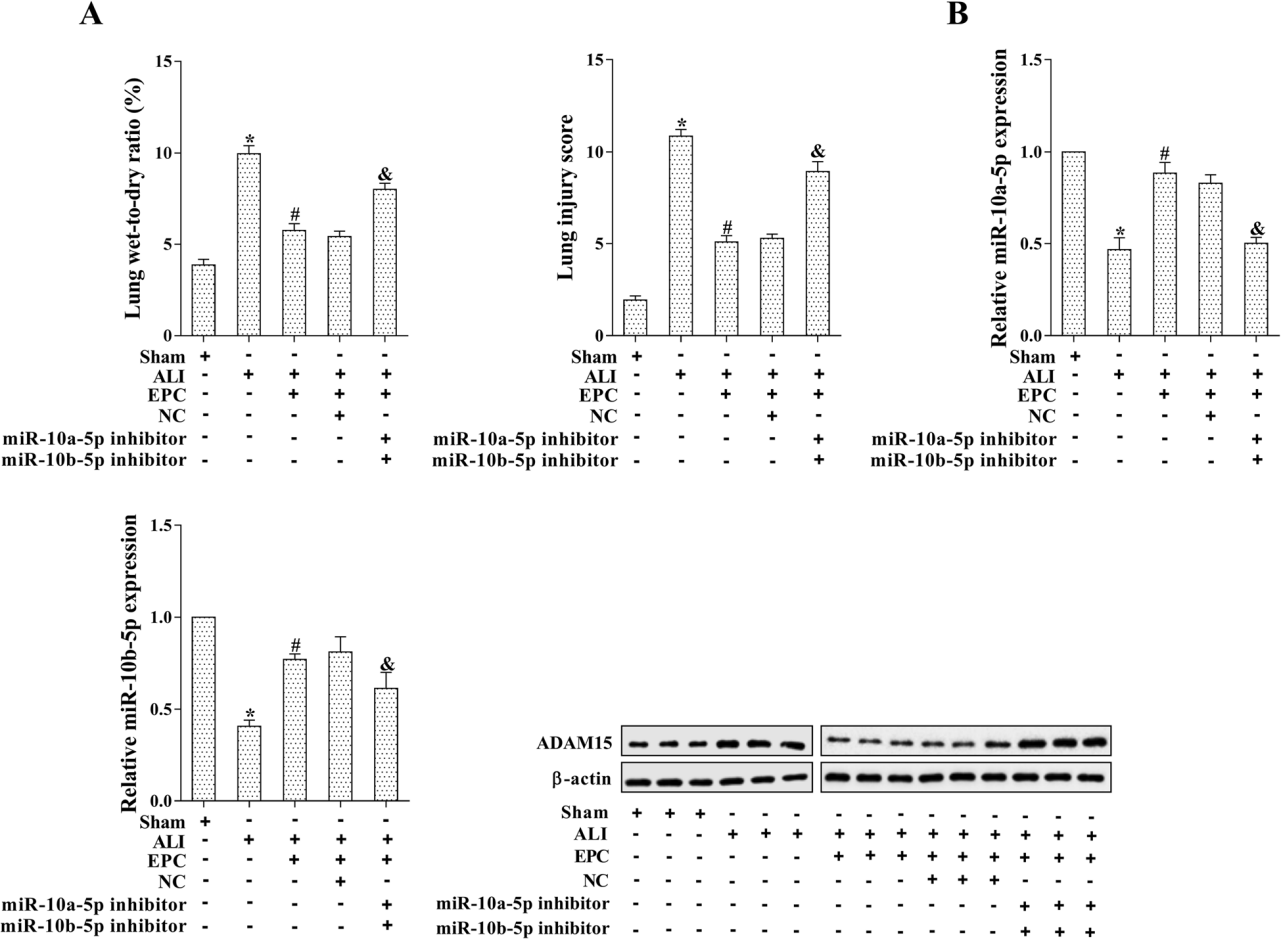
EPCs exerted the therapeutic effect of ALI via regulating miR-10a/b-5p/ADAM15. The IT administration of miR-10a-5p and miR-10b-5p inhibitors (2 mg/kg) was performed 24 h before the IT instillation of LPS and IV injection of EPCs. **a**: The level of lung wet-to-dry ratio and the average value of LIS. **b**: The expression of miR-10a/b-5p and ADAM15 was determined by qRT-PCR and western blot analysis. *p < 0.05 vs. sham; #*p* < 0.05 vs. ALI; & *p* < 0.05 vs. ALI + EPC + NC

Given the pro-proliferation effect of EPCs in LPS-induced MPMVECs, we explored the therapeutic effect of EPCs in the ALI mouse model. The IT administration of miR-10a-5p and miR-10b-5p inhibitors (2 mg/kg) was performed 24 h before the IT instillation of LPS and IV injection of EPCs. Both wet-to-dry ratio and LPS were restored by the down-regulation of miR-10a/b-5p, indicating that the therapeutic effect caused by EPCs was reversed by miR-10a/b-5p down-regulation (Fig. 6a, *p* < 0.05). Finally, qRT-PCR and western blot analysis confirmed the enhancement of miR-10a/b-5p after EPCs transplantation and the reduction of miR-10a/b-5p after miR-10a/b-5p down-regulation, as well as the corresponding negative-change of ADAM15 (Fig. 6b, *p* < 0.05). Taken together, these data demonstrated that EPCs exerted the therapeutic effect of ALI via increasing miR-10a/b-5p and reducing ADAM15.

## Discussion

EPC transplantation has been demonstrated to attenuate LPS-induced lung injury in animal models. However, little is known about the underlying mechanisms. In our study, we demonstrated that EPC transplantation exerted its therapeutic effect of ALI via increasing miR-10a/b-5p and reducing ADAM15. Our findings provided a novel insight into the molecular mechanism of EPC transplantation in treating ALI.

Endothelial dysfunction is the major pathological mechanisms of ALI, and the initiation of injury is mainly stimulated by microbial components [3]. These bacterial motifs, recognized by the innate immune system, are called pathogen-associated molecular patterns (PAMPs) [23]. LPS in Gram-negative bacteria is recognized as the endotoxin, which is embedded in the outer membrane of the bacterial cell wall. Unlike Gram-negative bacteria, there is no endotoxin in Gram-positive bacteria, but previous studies have identified some structural components that account for the biological activity [24, 25]. In the current study, we established the mouse ALI model by IT instillation of LPS, and the increased lung wet-to-dry ratio and LIS revealed the severe injury of lung tissues. Other bacterial components could be used to explore the mechanism of ALI in the future researches.

After the recognition of LPS and other bacterial components, endothelial cells will be activated and release some cytokines and pro-inflammatory factors to recruit leukocytes and to promote clotting [26]. During this process, endothelial cells may undergo necrosis or apoptosis because the tissue is reabsorbed and repaired. Therefore, promoting endothelial cell proliferation can help alleviate tissue injuries. In our work, we used LPS to induce cell injury of MPMVECs, and our findings demonstrated that EPC co-culture significantly increased cell proliferation, indicating EPC transplantation as an effective strategy of correct endothelial dysfunction. Other anti-apoptotic therapy includes activated protein C by down-regulating pro-apoptotic genes (calreticulin and TRMP-2) [27]. In addition, the maintenance of blood flow, early goal-directed therapy, and insulin has been demonstrated to inhibit cell apoptosis and promote endothelial cell survival [28–30].

EPCs are a class of cells that can differentiate into mature endothelial precursor cells, and play a role in blood vessel formation, the process of re-endothelialization, and repair after tissue injury. Recent studies have indicated that EPCs are involved in endothelial repairing and immune regulation, as well as in regulating vascular injury and inflammation [6, 7]. It was also reported that the number of peripheral blood EPCs is markedly increased in patients with pneumonia or sepsis, and the higher number of EPCs revealed a better prognosis [31]. Moreover, EPC transplantation has been demonstrated to attenuate LPS-induced lung injury in the animal models [12, 13]. Yang et al demonstrated that EPC transplantation significantly reduced lung, liver, and kidney tissue damage and wet-to-dry ratio by down-regulating TLR4 mRNA expression and reducing interleukin-6 (IL-6) and IL-10 levels [11]. In the current study, we transplanted EPCs into ALI mice, and found that the lung wet-to-dry ratio and LIS were significantly reduced, and such therapeutic effect was also seen in MPMVECs co-cultured with EPCs. Furthermore, the correlation between EPC and miR-10a/b-5p was confirmed by down-regulating miR-10a/b-5p expression. To this end, our findings indicated a new role of EPC transplantation, which is increasing the expression of miR-10a/b-5p.

In recent years, it has been reported that miRNAs can regulate the development and the response of the immune system, including antigen presentation, Toll-like receptor (TLR) signaling pathways, cytokines and lymphocyte receptor signaling. For example, the expression of miR-155 and miR-146 was up-regulated in immune response, and regulated the levels of nuclear factor-kappa B (NF-κB), interferon-γ (IFN-γ), tumor necrosis factor-α (TNF-α), and IL-1 via targeting genes [32, 33]. Dai et al demonstrated that the inhibition of miR-223 activity decreased LPS-induced IFNγ in splenic lymphocytes from estrogen-treated mice [34]. Xiao et al. proved that miR-150 regulated B cell differentiation by targeting the transcription factor c-Myb [35]. Additionally, miR-15a, miR-34a and miR-10a/b-5p were demonstrated to have aberrant levels in sepsis [18, 19], so we detected the different expressions of these miRNAs in ALI lung tissues. We chose miR-10a-5p and miR-10b-5p as the candidate miRNA according to the qRT-PCR results. With the confirmation of luciferase reporter assay and the overexpression and knockdown of miR-10a/b-5p, we proved that there is a target relationship between miR-10a/b-5p and ADAM15.

In conclusion, our findings demonstrated that EPCs increased the expression of miR-10a/b-5p and reduced ADAM15 protein level in LPS-induced ALI lung tissues and MPMVECs. Furthermore, EPC transplantation exerted its therapeutic effect of ALI via increasing miR-10a/b-5p and reducing ADAM15, thus providing a novel insight into the molecular mechanism of EPC transplantation in treating ALI. The importance of EPCs that affect inflammatory response has made them an appealing target for ALI therapy. Therefore, the clinical use of EPC transplantation in treating ALI deserves further investigations.

## Additional file

Additional file 1: **Figure S1.** The serum level of IL-6 (A) and TNF-α (B) was detected using ELISA assays. The pulmonary tissue level of HMGB1 (C) and SOD (D) was detected 48 h after EPCs transplantation. *p < 0.05 vs. sham; #*p* < 0.05 vs. ALI + PBS. (TIF 687 kb)

## Data Availability

The datasets used and/or analyzed during the current study are available from the corresponding author on reasonable request.
