# Supplementary figures and images for: Endothelial progenitor cell transplantation attenuates lipopolysaccharide-induced acute lung injury via regulating miR-10a/b-5p
Source: Lipids Health Dis. 2019 Jun 7;18:136. doi: 10.1186/s12944-019-1079-3 (PMC6556024; doi:10.1186/s12944-019-1079-3)

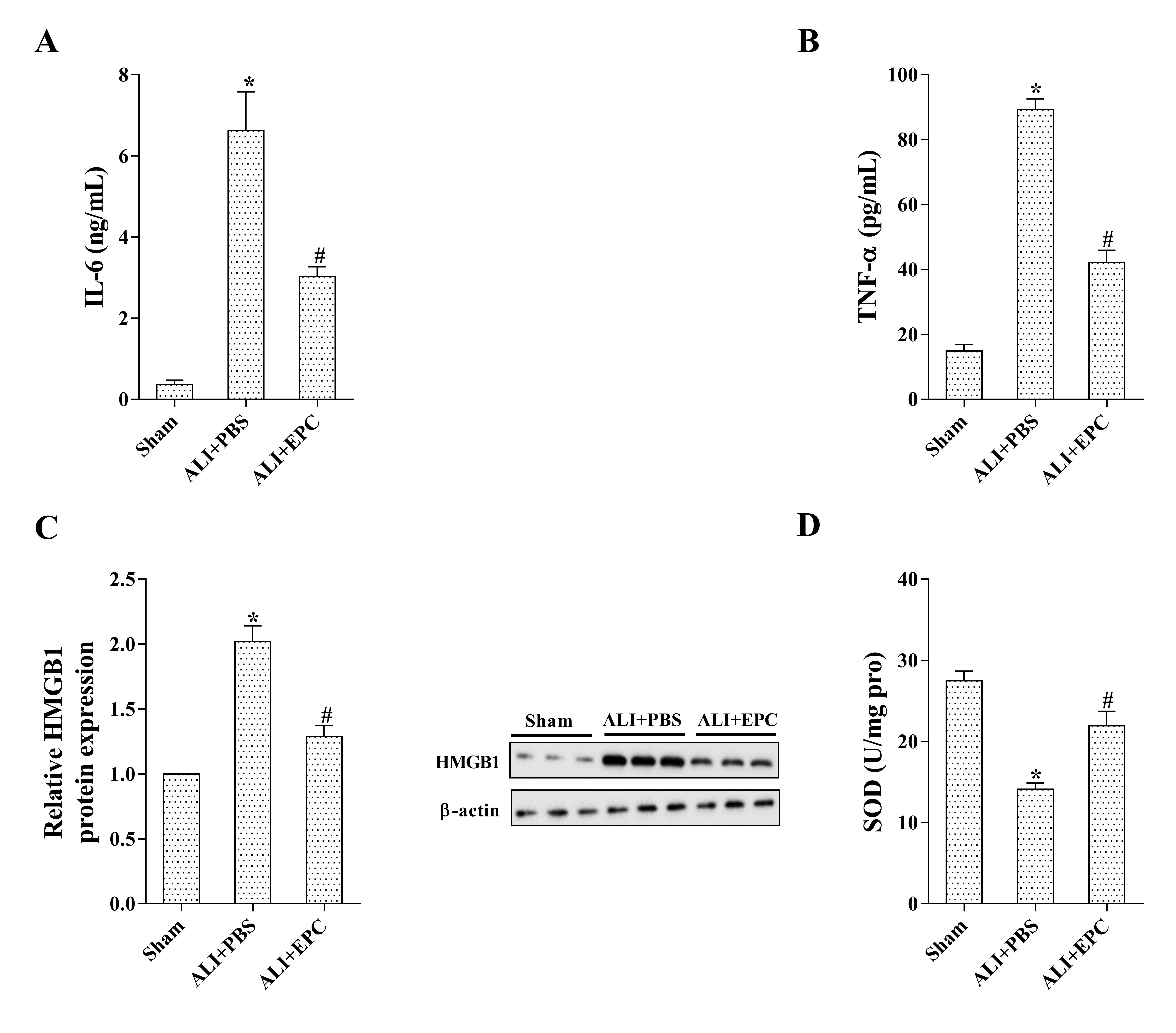

Supplement: Supplementary file 1 — Figure S1. The serum level of IL-6 (A) and TNF-α (B) was detected using ELISA assays. The pulmonary tissue level of HMGB1 (C) and SOD (D) was detected 48 h after EPCs transplantation. *p < 0.05 vs. sham; #p < 0.05 vs. ALI + PBS. (TIF 687 kb) [file 12944_2019_1079_MOESM1_ESM.tif]
